# Early raise of BDNF in hippocampus suggests induction of posttranscriptional mechanisms by antidepressants

**DOI:** 10.1186/1471-2202-10-48

**Published:** 2009-05-13

**Authors:** Laura Musazzi, Annamaria Cattaneo, Daniela Tardito, Alessandro Barbon, Massimo Gennarelli, Sergio Barlati, Giorgio Racagni, Maurizio Popoli

**Affiliations:** 1 Center of Neuropharmacology – Department of Pharmacological Sciences and Center of Excellence on Neurodegenerative Diseases, University of Milan, Milan, Italy; 2 IRCCS, FBF S. Giovanni di Dio, Brescia, Italy; 3 Division of Biology and Genetics, Department of Biomedical Sciences and Biotechnologies, University of Brescia, Brescia, Italy

## Abstract

**Background:**

The neurotrophin BDNF has been implicated in the regulation of neuroplasticity, gene expression, and synaptic function in the adult brain, as well as in the pathophysiology of neuropsychiatric disorders and the mechanism of action of antidepressants. Antidepressant treatments have been shown to increase the expression of BDNF mRNA, although the changes measured were found to be different depending on various factors. A few studies only have measured levels of BDNF protein after antidepressant treatments, and poor correlation was found between mRNA and protein changes. We studied the time course of expression of BDNF mRNA and protein during drug treatments, in order to elucidate the temporal profile of regulation of this effector and whether mRNA and protein levels correlate. Rat groups were treated for 1, 2 or 3 weeks with fluoxetine or reboxetine; in additional groups drug treatment was followed by a washout week (3+1). Total BDNF mRNA was measured by Real Time PCR, pro- and mature BDNF proteins were measured by Western blot.

**Results:**

We found that mature BDNF protein is induced more rapidly than mRNA, by both drugs in hippocampus (weeks 1–2) and by reboxetine in prefrontal/frontal cortex (week 1). The temporal profile of BDNF protein expression was largely inconsistent with that of mRNA, which followed the protein induction and reached a peak at week 3.

**Conclusion:**

These results suggest that BDNF protein is rapidly elevated by antidepressant treatments by posttranscriptional mechanisms, and that induction of BDNF mRNA is a slower process.

## Background

Brain-Derived Neurotrophic Factor (BDNF) is an abundant neurotrophin regulating neuroplasticity, gene expression, synaptic function and cognition in the adult brain [1,2], that has been implicated in the pathophysiology of various neuropsychiatric and neurodegenerative disorders as well as in the mechanism of action of antidepressant drugs [3-9]. It is generally assumed that antidepressant treatments increase the expression of BDNF, which, according to the neurotrophic hypothesis, represents an effector of changes in neuroplasticity and cellular resilience mediating the long-term therapeutic effect of antidepressants. In this framework BDNF has lately become, together with the activation of cAMP-response element binding protein (CREB), a sort of readout system in the study of antidepressant mechanisms [4,5,7,8,10-14].

However, several studies have shown that the changes in the expression of BDNF can be quite different depending on various factors, such as type of drug, dosage, route of administration, length of treatment (see ref. [9] for discussion). Moreover, only a few studies have measured levels of BDNF protein after antidepressant treatments [10,15-19] and, when mRNA and protein levels have been measured at the same time, poor correlation was found between mRNA and protein changes [16,17]. In addition, the rat BDNF gene has a complex structure with at least eight 5' exons that can be spliced to a single 3' exon containing the coding domain for the BDNF protein, generating 11 different transcripts according to the last nomenclature [20]. This makes the effect of drug treatments on BDNF expression more complex to explain, because the changes in total BDNF can potentially be related to changes in different non-coding exons spliced to the coding exon (exon IX).

In this study, with regard to the action of antidepressants, we asked the following questions:

1. What is the time course of BDNF expression during antidepressant treatment? Is it consistent with the onset of therapeutic effect?

2. What is the expression level of BDNF after1 week-washout?

3. How does BDNF protein expression profile during treatment correlate with BDNF mRNA expression?

In order to assess the time-course of BDNF expression during long-term antidepressant treatments, we have treated rats with two different drugs endowed with complementary mechanisms: fluoxetine (FLX), a selective serotonin reuptake inhibitor (SSRI), and reboxetine (RBX), a selective norepinephrine reuptake inhibitor (NRI). The drug treatments were carried out for 1, 2 or 3 weeks and were followed by an additional washout week (3+1), that was added in order to study the fate of BDNF expression when antidepressant treatment is discontinued. In all these rat groups we assessed the expression of total BDNF at both mRNA and protein levels, measuring both the pro- and mature forms of BDNF [1,21,22]. We found that mature BDNF protein is induced in hippocampus more rapidly than mRNA, suggesting that antidepressants rapidly regulate BDNF at posttranscriptional level.

## Results

### Distinct temporal profile of expression of total BDNF mRNA induced by fluoxetine and reboxetine

**Figure 1:**
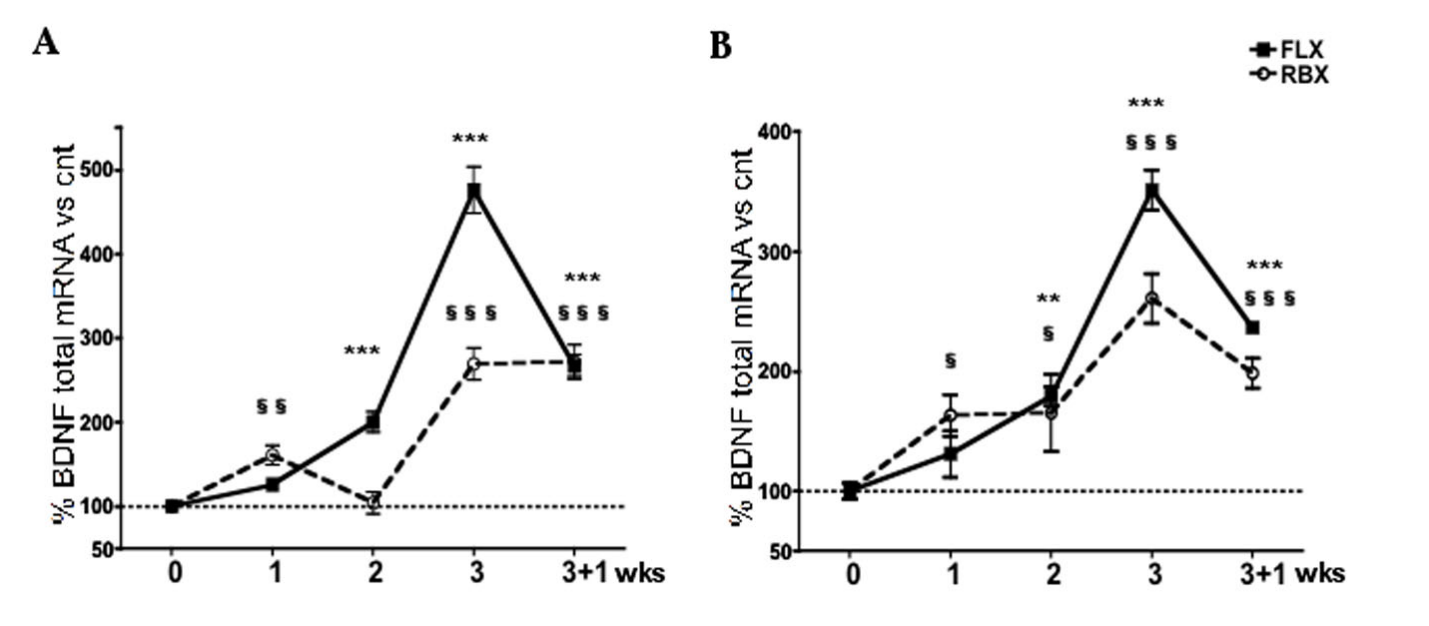
**Time course of expression levels of BDNF total mRNA following antidepressant treatment**. Expression levels of BDNF total mRNA in HPC (A) and P/FC (B) of rats treated with FLX and RBX. Data are expressed as % of controls (mean ± s.e.m.). Bonferroni post-hoc test: FLX-treated vs. control rats: ** p < 0.01, *** p < 0.001; RBX-treated vs. control rats: § p < 0.05, §§ p < 0.01, §§§ p < 0.001.

We measured the changes in BDNF expression induced by the two drug treatments at the level of both mRNA and protein, in HPC and P/FC. Total BDNF mRNA was detected by means of quantitative Real Time PCR, using primers designed on the sequence of the coding exon (exon IX). 2-Way ANOVA showed in HPC an effect of time (F_4,79 _= 147.31; p < 0.0001), drug (F_1,79 _= 37.31; p < 0.0001) and time/drug interaction (F_4,79 _= 24.61; p < 0.0001). Similarly, in P/FC there was an effect of time (F_4,50 _= 44.24; p < 0.0001), drug (F_1,50 _= 4.38; p < 0.0001) and time-drug interaction (F_4,50 _= 3.80; p < 0.01). As shown in Fig. 1A–B, the temporal profile of activation of mRNA expression by FLX and RBX was similar in the two brain areas, but typical of either drug. The levels of total BDNF mRNA were induced starting with week 1 (RBX) or 2 (FLX) and peaked at week 3 with both drugs (particularly with FLX). During the washout week the mRNA level was sharply reduced for FLX, although maintaining a significantly higher than basal level. This gap between week 3 and 3+1 was less pronounced for RBX, for which the mRNA level at week 3+1 was also significantly higher than basal level.

### Expression profile of BDNF protein induced by fluoxetine and reboxetine consistent with early translation of mRNA

**Figure 2:**
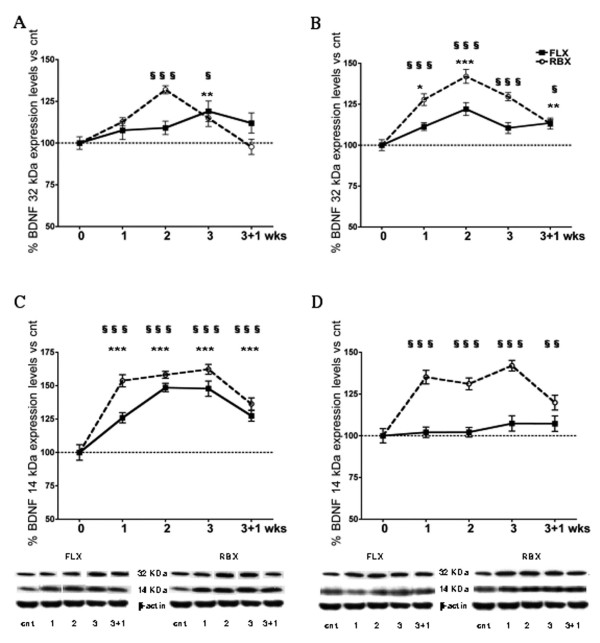
**Time course of expression levels of BDNF protein following antidepressant treatment**. Time course of expression levels of BDNF protein precursor of 32 kDa (A, B) and mature protein of 14 kDa (C, D) in HPC (A, C) and P/FC (B, D) of rats treated with FLX or RBX. Insets: representative immunoreactive bands from Western blots. Data and statistic analysis as above. FLX-treated vs. control rats: * p < 0.05, ** p < 0.01, *** p < 0.001; RBX-treated vs. control rats: § p < 0.05, §§ p < 0.01, §§§ p < 0.001.

The expression of the BDNF protein follows a multi-step process of maturation from a pre-propeptide through a proprotein to the mature form of BDNF [21]. We measured by Western blot both the 32 kDa pro-BDNF and 14 kDa mature BDNF, the two most abundant forms of the protein [1,5], in the total extract of HPC and P/FC at the different treatment times as above (Fig. 2). The two drugs showed different effects on BDNF protein levels in the two areas. In HPC (Fig. 2A–C), 2-Way ANOVA showed an effect of time and drug for both pro-BDNF (F_4,59 _= 26.71; p < 0.0001; F_1,59 _= 24.16; p < 0.0001) and mature BDNF (F_4,89 _= 12.17; p < 0.0001; F_1,89 _= 65.08; p < 0.0001) and of time/drug interaction for pro-BDNF (F_4,59 _= 4.65; p < 0.01). In HPC, FLX induced a trend toward increase of pro-BDNF at weeks 1–2, which became significant at week 3, while RBX up-regulated pro-BDNF at weeks 2–3. Both FLX and RBX consistently up-regulated the expression of mature BDNF at all times in HPC, with maximum at weeks 2–3 for FLX and 1 to 3 for RBX (Fig. 2A–C).

In P/FC (Fig. 2B–D), 2-Way ANOVA showed an effect of time (F_4,53 _= 7.69; p < 0.0001) and time/drug interaction (F_4,53 _= 4.07; p < 0.01) for pro-BDNF and an effect of time (F_4,54 _= 44.79; p < 0.0001) and drug (F_1,54 _= 12.53; p < 0.001) for mature BDNF. Indeed, both FLX and RBX up-regulated pro-BDNF, with the only exception of week 3 for FLX. Differently from the HPC, in P/FC the up-regulation of pro-BDNF by FLX sharply contrasted with the absence of changes in mature BDNF (Fig. 2B–D). As in HPC, RBX consistently up-regulated mature BDNF at all times.

Overall, the regulation of mRNA and protein was not consistent in HPC and P/FC for both drugs. No mature BDNF was induced by FLX in P/FC. The results showed that in HPC the level of mature BDNF protein reached a peak (weeks 1–2) before the actual peak of mRNA (week 3), suggesting early translation and processing of the protein during antidepressant treatment. The same was observed for RBX in P/FC.

## Discussion

The main findings of this work were that BDNF mRNA and protein levels during antidepressant treatments did not correlate, and that induction of mature protein preceded that of mRNA. The measurement of BDNF mRNA expression showed an overall similar temporal profile of induction with the two drugs. RBX acted somewhat faster, inducing BDNF mRNA already at week 1 in both areas, while FLX induced BDNF from week 2. The shape of this induction was also somewhat different; FLX progressively increased the expression up to week 3, while RBX induced BDNF in two distinct waves. After a precocious induction at week 1, the expression of BDNF was not further increased (P/FC) or was reduced (HPC) by RBX between week 1 and 2. A second, more pronounced, wave of BDNF induction by RBX was observed between week 2 and 3. Overall, RBX acted faster on BDNF transcription but FLX outsized RBX with regard to the maximum extent of mRNA induction. Interestingly, the washout week also differently affected the outcome of the two drug treatments in HPC, because it sharply reduced the effect of FLX in both areas and of RBX in P/FC, while in HPC of RBX-treated rats BDNF mRNA level was unchanged at week 3+1. In a companion study [23], we previously found that 1 washout week entirely abolished the activation of CREB induced by 3 weeks of RBX, but not of FLX, in line with the longer half-life of norFLX, the active FLX metabolite. This discrepancy between the effects of washout on activation of CREB vs. BDNF expression may underscore the complex regulation of the BDNF gene, that contains several different regulatory elements (other than CRE) at the level of promoters [24].

Surprisingly, the temporal profile of BDNF protein expression in HPC was largely inconsistent with that of mRNA expression (compare Fig. 1A with Fig. 2C). Although with FLX a significant increase of mRNA was not observed until week 2, in the same rats mature BDNF (14 kDa) was already significantly elevated at week 1 and reached maximal level at week 2, when mRNA level was still far below the maximal level attained at week 3. Instead, the level of pro-BDNF (32 kDa) in HPC was significantly elevated only at week 3. A possible explanation for this discrepancy is that, in the first several days of FLX treatment, BDNF mRNA already available is rapidly translated and the proBDNF produced is also rapidly processed to mature BDNF, suggesting that FLX has a more rapid effect on BDNF translation/processing than on the transcription of BDNF gene. Although more in line with the early induction of mRNA by RBX, the profile of induction of mature BDNF by this second drug was also inconsistent with the profile of mRNA induction, which reached maximal level only at week 3 (as for FLX). With RBX the level of mature BDNF was already maximal at week 1 and remained steadily elevated up to week 3, suggesting again a rapid effect of the drug on BDNF translation/processing, perhaps accelerated by the induction of new mRNA at week 1 by RBX.

In P/FC the effects of the two drugs on BDNF translation were different from each other. Maximal level of mature BDNF was induced by RBX already at week 1, as in the HPC, although the extent of increase was somewhat smaller than in HPC. Again, the temporal profile of the protein was inconsistent with that of mRNA, which reached maximal level only at week 3. By contrast, no mature BDNF was detected at any time point during FLX treatment, although moderate levels of proBDNF were attained, suggesting that in P/FC FLX was able to induce transcription of BDNF but little translation and no processing of the protein. Previous studies did not find induction of BDNF protein by FLX or other antidepressants in frontal cortex [15,17]. This substantial lack of induction of BDNF protein by FLX in P/FC would suggest that downstream effects of this drug on synaptic plasticity are more powerful in HPC than in prefrontal/frontal areas of cerebral cortex.

The present results are in line with the several reports that previously showed up-regulation of BDNF mRNA induced by chronic antidepressant treatments, often increasing with the length of treatment [16,25]. We also found here, as in two previous studies [16,17], a lack of correlation between mRNA and protein, to the point that in HPC detectable levels of BDNF protein were raised faster than those of mRNA by the drug treatment. Overall, the differences in levels of BDNF protein we detected are somewhat bigger than in previous studies using the same drugs; this could be due to the fact that we measured here for the first time BDNF levels in a time-course treatment by Western analysis rather than by ELISA, a method that does not allow to distinguish between pro- and mature BDNF.

The present results would suggest that two antidepressants, FLX and RBX, have a particularly fast effect on posttranscriptional regulation of BDNF in HPC (RBX also in P/FC). It has already been suggested that BDNF synthesis could be regulated at posttranscriptional level [17], as found for other proteins [26]. First, it is known that selected BDNF transcripts are located at dendrites, a mechanism that allows rapid regulation of translational control [27]; we recently found that in rats treated with FLX or RBX more BDNF mRNA is located at dendrites in CA3 hippocampal area (Tongiorgi E, Popoli M; unpublished). Second, it was recently suggested that microRNAs exert a robust effect on BDNF levels in mature human prefrontal cortex, participating in posttranscriptional fine tuning of BDNF expression [28]. Third, it was recently shown that cleavage of pro- to mature BDNF is regulated by neuronal activity [22]; changing the processing of pro-BDNF by proteolytic enzymes could be an additional way whereby antidepressants rapidly increase the production of mature BDNF. This could explain why in HPC we found a faster rise of mature BDNF compared to pro-BDNF (compare Fig. 2A with Fig. 2C). Investigation of these mechanisms in subchronic and chronic treatments with antidepressants is warranted.

Finally, the finding that BDNF, a major readout effector, is rapidly elevated in HPC by antidepressant treatment may suggest that later downstream events are required to elicit systemic and behavioral effects of antidepressants. If BDNF production is faster than thus far envisaged, perhaps the correlates of the therapeutic effects should be searched in the actual cellular/molecular changes induced by the action of BDNF, such as the modifications in synaptic plasticity and the increment of neurogenesis. Further work is required to characterize these events.

## Conclusion

The results of the present study show that BDNF protein is rapidly elevated by antidepressant treatments whereas the induction of BDNF mRNA is a slower process, suggesting that posttranscriptional mechanisms are involved in the mechanism of antidepressants.

## Methods

### Drug treatment and preparation of total extract

**Figure 3:**
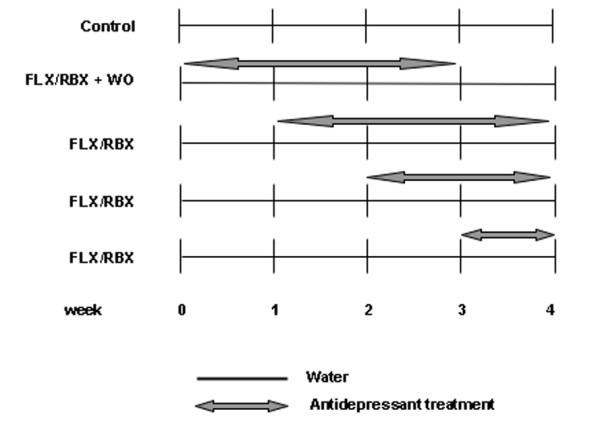
**Schematic representation of treatment time course for the different rat groups**. The arrows indicate the length of treatment with fluoxetine (FLX) or reboxetine (RBX) in weeks. The control group received only water for 4 weeks. WO: washout. All animals were sacrificed at the end of the 4^th ^week.

Experiments complied with guidelines for use of experimental animals of European Community Council Directive 86/609/EEC. All efforts were made to minimize animal distress and to reduce the numbers of animals used in this study. Groups of 12 male Sprague-Dawley rats (6 week-old at beginning of treatment) (Charles River Calco, Italy) were treated with vehicle (water), FLX or RBX at 10 mg/kg rat weight/day, delivered in drinking water; the average water intake per day for each cage (two rats) was recorded for 4 days before starting and throughout the treatment, and the drug solutions were changed every two days according to animals' weight, as reported in previous studies [29]. Rats were divided in 9 experimental groups: control (water), 1 week-treatment (FLX or RBX), 2 week-treatment (FLX or RBX), 3 week-treatment (FLX or RBX) and 3 week-treatment (FLX or RBX) plus 1 week drug washout (Fig. 3). All the animals were sacrificed at the same age. Hippocampus (HPC) and the whole frontal lobe, referred to as prefrontal/frontal cortex (P/FC), were quickly excised on ice as previously shown [30,31]. Each hemi-HPC and -P/FC from right or left hemisphere was taken separately and randomly assigned for RNA (frozen) or protein analysis. For the latter, the tissue was freshly homogenized 1:10 (w/v) in homogenization buffer (0.28 M sucrose, 10 mM HEPES pH 7.4, 0.1 mM EGTA, 20 mM NaF, 5 mM Na_2_PO_4_, 1 mM Na_2_VO_4_, 2 ml/ml of protease inhibitor cocktail (Sigma-Aldrich, St Louis, MO) to obtain the total extract, that was aliquoted and stored at -80°C.

### RNA isolation, cDNA synthesis and Real-time quantitative PCR

Total RNA was isolated with Trizol (Invitrogen, Carlsbad, CA). 2 μg of RNA were reverse transcribed using SuperScript™ II Reverse Transcriptase (Invitrogen) and 0.5 μg random hexamers (Promega, Madison, WI) in a 50 μl final volume. Real-time PCR was carried out using a LightCycler rapid thermal cycler System (Roche Diagnostics, Mannheim, Germany) and SYBR Green was used for the detection of double-strand DNA. The PCR reaction was set up into microcapillary tubes in a volume of 20 μl with 2 μl of cDNA and 2 μl of LightCycler DNA Master SYBR Green I (Roche Diagnostics). For BDNF quantitation, fwd AGCTGAGCGTGTGTGACAGT and rew ACCCATGGGATTACACTTGG primers were used, while for β-actin quantitation, used as internal control [32,33], fwd GGGAAATCGTGCGTGACATT and rwd CGGATGTCAACGTCACACTT primers were used. The PCR program included an initial denaturation step during 30 s at 95°C, followed by 45 cycles of a 1 s melting step at 95°C, a 10 s annealing step at 58°C, and a 10 s elongation step at 72°C (all temperature transition rates were 20°C/s). At the end of each cycle, the fluorescence emitted by SYBR Green was measured. At the end of PCR reaction, samples were subjected to a temperature ramp (from 70°C to 95°C, 2°C/s) with continuous fluorescence monitoring for melting curve analysis. For each PCR product, a single narrow peak was obtained by melting curve analysis at the specific temperature. Each sample was assayed in duplicate and the analysis was performed with Light Cycler Relative Quantification Software. Samples containing no template were used as negative controls in each experiment.

### Western blot Analysis

Western blot analysis was carried out as described previously [34], by incubating PVDF membranes, containing electrophoresed proteins from total homogenates, with polyclonal antibodies for BDNF 1:500 (Santa Cruz Biotechnologies, Santa Cruz, CA). A minimum of 2–3 replicates per rat were carried out in the different treatment groups (vehicle, FLX, RBX). Following incubation with peroxidase-coupled secondary antibodies, protein bands were detected by using ECL (GE Healthcare, Uppsala, Sweden). Standard curves were obtained by loading increasing amounts of samples on gels as described previously [34]. All protein bands used were within the linear range of standard curves and normalized for β-actin level in the same membrane. Standardization and quantitation was as reported previously, except that Quantity One software (BioRad Laboratories, Hercules, CA) was used.

### Statistical Analysis

All data were analyzed by using two-way analysis of variance (2-Way ANOVA) for the variable treatment (FLX/RBX) and the variable time (Cnt, 1 week, 2 weeks, 3 weeks, 3+1 washout week). If the analysis of variance revealed significant group differences, post-hoc Bonferroni tests were carried out to elucidate the pattern of group differences. Significance was assumed at p < 0.05. Statistical analysis of the data was carried out using GraphPad Prism 4 (GraphPad Software, Inc., San Diego, CA).

## Authors' contributions

LM carried out the animal treatments, the western analysis and performed the statistical analysis. AC carried out the molecular biology work. DT performed the statistical analysis, and drafted the manuscript. AB carried out the molecular biology work. MG planned the experiment for BDNF RNA transcription. SB helped to draft the manuscript and provided useful discussion. GR helped to draft the manuscript and provided useful discussion. MP designed the study, and drafted the manuscript. All authors contributed to and have approved the final manuscript.
